# Obesity Associated Molecular Forms of C-Reactive Protein in Human

**DOI:** 10.1371/journal.pone.0109238

**Published:** 2014-10-09

**Authors:** Bela F. Asztalos, Michael S. Horan, Katalin V. Horvath, Ann Y. McDermott, Naga P. Chalasani, Ernst J. Schaefer

**Affiliations:** 1 Lipid Metabolism Laboratory, Human Nutrition Research Center on Aging at Tufts University, Boston, Massachusetts, United States of America; 2 Indiana University School of Medicine, Indianapolis, Indiana, United States of America; Jilin University, China

## Abstract

**Objective:**

To describe novel C-reactive protein (CRP) molecular forms (mf) in human plasma.

**Design and Methods:**

Five novel CRP-mfs, disctinct from the previously described native (nCRP) and modified (mCRP) C-reactive proteins, were separated from human plasma by polyacrylamide gel electrophoresis and immunodetected by western blot in subjects with or without increased BMI, cardiovascular disease (CVD), and diabetes (n = 1800).

**Results:**

Three of the five CRP-mfs were present in all samples. One, CRPmf-4, was present in a subgroup of subjects and its presence was associated with elevated body mass index (BMI). CRP-mf-5 was present in about 2% of the subjects and was not associated with any other parameters. The presence or distribution of the 5 CRP-mfs were not Ca^2+^-dependent. Crossed immuno-localization experiments indicated that none of the CRP-mfs were complexed with any of the lipoprotein classes or with signature proteins of the complement-factor. Moreover, the distribution of CRP-mfs were not significantly correlated with plasma CRP levels. CRP-mf-4 was significantly associated with increased BMI, but not with other parameters of the metabolic syndrome (HDL-C and triglyceride levels, and diabetes).

**Conclusions:**

We have identified five new CRP-mfs out of which CRP-mf-4 was significantly associated with obesity. We have shown that oligomerization of CRP was not calcium dependent. We hypothesize that adipose tissue produces a factor which influences the formation of CRP mf-4. CRP-mfs might be used as an obesity-associated inflammatory marker.

## Introduction

C-reactive protein (CRP) is a sensitive systemic marker of inflammation and has been shown to be an independent predictor of coronary heart disease (CHD) risk. CRP contains 224 amino acids, with a molecular weight of 25KD for the monomeric form. The human *CRP* gene is located on chromosome one (1q21-q23). In humans, 5 CRP proteins form an annular pentameric disc which places CRP into the pentraxins protein class [1]. CRP is a member of the class of acute-phase proteins whose levels rise dramatically during inflammatory processes occurring in the body. This increment is due to a rise in the plasma concentration of interlukin (IL)-6, which is produced predominantly by macrophages [2] as well as adipocytes [3]. CRP assists in complement binding to foreign and damaged cells and enhances phagocytosis by macrophages which express a receptor for CRP. CRP may play another important role in innate immunity, as an early defense system against infection.

CRP is also a sensitive marker of inflammation and tissue damage [4] in the vasculature and can predict acute coronary events [5]–[7]. CRP as a cardiovascular disease (CVD) risk marker has been in the focus of interest lately, especially with the findings of the JUPITER trial. The JUPITER investigators reported a 37% decrease in CRP levels during the statin (rosuvastatin) trial [8]. Decreases in major CVD events were observed in subjects with elevated CRP, but healthy otherwise; Framingham risk score <10% and all lipid parameters below the threshold for intervention.

It is documented that native CRP (nCRP) and modified CRP (mCRP) are different in their physiologic functions [9]–[11]. A German group reported that nCRP and mCRP have opposite effects on atherosclerosis in apoE^-/-^ mice [10]. A Canadian group reported that mCRP, but not nCRP, promotes a proinflammatory human endothelial-cell phenotype [12]. Our group has identified several new CRP molecular forms (CRP-mf), separated by native polyacrylamide gel electrophoresis (PAGE) and detected by immunoblotting. In this study, we describe the method for separating CRP-mfs from whole plasma and the associations of CRP mf-4 with obesity.

## Methods

### Subjects

Plasma samples selected for this study were from: 1) the Framingham Offspring Study (FOS) cycle 6 (500 male and 300 female participants without a history of CVD and 263 male and 61 female participants with a history of CVD; 2) the Veterans Affairs HDL Intervention Trial (VA-HIT) (600 males with established CVD and low HDL-C level); and 3) studies on obesity: 60 morbidly obese subjects with a BMI≥40 kg/m^2^ of whom 20 underwent gastric bypass surgery and whose samples were collected before and after the procedure.

All research was approved by the Institutional Review Board at Tufts Medical Center and Tufts University Health Sciences Campus and by the Institutional Review Board at Indiana University Purdue University at Indianapolis. Work was done using deidentified samples from our blood banks; therefore, exception-4 IRB approvals were received.

### Methods

CRP molecular forms (CRPmf) were determined by 1-dimensional non-denaturing PAGE. 20 µl of plasma were applied to 3–16% linear-gradient gels and run in chilled (7°C) Tris-Borate buffer at 250 V for 4 hrs. Gels were electro-transferred to nitrocellulose membranes in chilled (7°C) Tris-Glycine buffer followed by immuno-detection with monospecific CRP antibodies [goat anti human pab, (in-house and Biodesign International Saco, Maine)]. In selected samples, monoclonal antibodies against mCRP (clone-9C9 and clone-C6 from Meridian Life Science and clone-8 from Sigma) were used. Purified native (nCRP) and modified (mCRP), used as standard in electrophoreses, were purchased from Sigma and were tested by SDS gel electrophoresis under reducing and non-reducing conditions.

The signal of the ^125^I-labeled secondary antibody was quantitatively evaluated by a Storm 860 image analyzer (Molecular Dynamics, CA) and ImageQuant software. CRP-mf distributions were calculated as percentile of the total signal and if needed, these percentiles were multiplied with plasma CRP concentration for calculating absolute values of the individual CRP-mfs. A 5% cut point was used to identify the presence of a CRP-mf on the electrophoretogram (i.e. the CRP-mf had> 5% of the total amount of CRP present in different CRP-mfs).

### Statistical analysis

ANOVA was used for estimating associations between CRP-mfs and other biomedical parameters. Student's t-test was used to calculate associations between changes in the concentration of CRP-mfs and other biomedical parameters.

## Results

### Separation of CRP from whole plasma by gel electrophoresis

Our goal was to test whether the 2 known CRP isoforms, native/pentameric (nCRP) and modified/monomeric (mCRP), were associated with low density lipoprotein (LDL), high density lipoprotein (HDL), and CVD.

We have used 2 different monospecific polyclonal antibodies against nCRP and 3 different monospecific antibodies against mCRP. Using SDS PAGE, we have verified that the 2 types of antibodies were monospecific to nCRP and mCRP.

**Figure 1:**
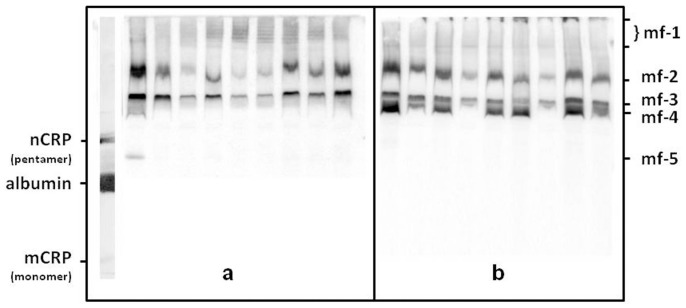
CRP-mfs were separated on 3–16% linear gradient PAG. Electrophoreses were carried out under non-denaturing conditions. Gels were electrotransfered to nitrocellulose membrane followed by imuno-probing membranes with a polyclonal antibody specific to human CRP. Subjects' total plasma CRP values varied between less than 2 µg/mL (columns 4, 6, 7 in panel-a, and columns 4 and 7 in panel-b) and about 20 µg/mL (column 2 in panel-a, and column 1 panel-b). Column 1 in panel a shows the native or pentamer (nCRP) and the modified or monomer (mCRP) as well as the human serum albumin. It is worth noting that under non-denaturing conditions proteins are separated by their mass/charge ratio not by size.

To test whether CRP attaches either to LDL or HDL in the circulation, we separated lipoproteins by 2-dimensional non-denaturing agarose/PAGE, followed by electrotransfer to nitrocellulose membrane and immuno-blotted for apoB, apoA-I, and CRP. We detected very low CRP signals which were close to the position of the sample load on the agarose gel. Further investigation indicated that under these experimental conditions, CRP bound to agarose and had very limited mobility and could not be electro-transferred to the nitrocellulose membrane. Therefore, we decided to separate lipoproteins by 1-dimensional non-denaturing PAGE. By cross-immuno detection, we have observed a portion of CRP migrating in the size range of LDL and large HDL particles. Although in close proximity, both the apoB and apoA-I peaks were clearly diverse form CRP peaks indicating an incidental co-migration rather than an association between CRP and LDL and HDL particles. These experiments provided no proof of associations between CRP and lipoproteins but the data clearly showed multiple bands of CRP in all samples (**Figure 1**). Therefore, the next logical step was to test whether these CRP bands were similar to the known nCRP and mCRP. None of the anti mCRP antibodies produced signals when plasma was separated by non-denaturing 1-dimensional electrophoresis indicating that there was no detectable amount of mCRP in human plasma under these conditions. Moreover, the mCRP control was significantly further in the gel and did not co-migrate with the bands recognized by the polyclonal antibodies. In contrast, the 2 nCRP antibodies gave strong and identical patterns when plasma was separated under the same conditions.

We named these previously undescribed bands CRP molecular forms (CRP-mfs). We then tested if CRP-mfs were sensitive to Ca^2+^ concentration. We subjected serum and plasma samples from different types of blood collection tubes -serum, EDTA, citrate, and heparinized- to PAGE. Plasma and serum samples from the same subject gave us similar patterns indicating that CRP-mfs were not Ca^2+^ dependent. We also ran samples as fresh and frozen -after an -80°C freeze-thaw cycle- and saw no difference in the separation. Next, we did co-immunoaffinity precipitation and immunaffinity column chromatography using either immobilized polyclonal antibody against CRP or immobilized phosphocoline for purifying CRP from whole plasma. Any attempt for purifying CRP from plasma resulted in the loss of the various molecular forms even when physiological levels of Ca^2+^ and NaCl were maintained during the procedures.

Since associations with lipoproteins did not explain the various CRP-mfs we have checked whether the CRP bands co-migrated with some of the complement-proteins (IgG, IgM, C3, C4a, C4b, and Clusterin) in human plasma by cross-immunoblotting the membranes. None of the complement proteins co-migrated with CRP-mfs indicating that CRP in circulating human plasma was not complexed with these complement-factors. We also checked whether serum amyloid-P (SAP), another pentraxin in human plasma, was complexed with CRP, but found no SAP-CRP complex to be present in human plasma.

### Physiological characterization of CRP-mfs

Since CRP concentrations are associated with CVD risk, we tested whether CRP-mfs were also associated with this risk. We have used samples from 2 well-characterized cohorts for this purpose: the Framingham Offspring Study (FOS) and the Veterans Affairs HDL Intervention Trial (VA-HIT). We separated CRPmfs in 1124 samples from the FOS and in 600 samples from the VA-HIT. The major characteristics of the subjects are described in Table 1. We have observed differences in the distribution of CRP-mfs in apparently healthy male subjects from the FOS and in CHD patients from the VA-HIT (Table 2). In various percentiles, 3 of the molecular forms (CRPmf-1, CRPmf-2, and CRPmf-3) were present in practically all subjects. CRPmf-4 was present in about 60% and CRPmf-5 was present only in about 2% of subjects (Table 2 and Figure 1). Some other forms were also present in <1% of subjects. CRP-mf-1 included several similar intensity bands close to the origin overlapping the VLDL/LDL size range. CRP-mf-2 and CRP-mf-3 run faster on the gel and appeared in the HDL-size range. In the vast majority of cases, CRP-mf-2 and CRP-mf-3 were the dominant CRP forms. CRP-mf-4 had slightly higher electrophoretic mobility than CRP-mf-3 and its concentration varied- independent of total CRP concentration or those of other CRP-mfs. CRP-mf-5 had the highest electrophoretic mobility and was detected above the ingenious albumin and close to the nCRP standard indicating that CRP-mf-5 could be a pentamer form of CRP with a 125 KD molecular size.

**Table 1:** Characteristics of subjects included in the analyses.

	FOS male		FOS female		VA-HIT		Obese Group (n = 60)
	No CHD (n = 500)	CHD (n = 263)	No CHD (n = 300)	CHD (n = 61)	No Rx (n = 300)	Rx (n = 300)	
Age (Year)	58±10	65±8	58±9	67±8	64±7	64±7	44±8
BMI (kg/m^2^)	28±5	29±7	28±4	30±7	29±4	29±5	42±6
CRP* (mg/L)	1.9	2.6	1.9	2.3	3.3	3.4	6.7
TC (mg/dL)	192±30	186±35	212±40	215±36	168±25	158±30	189±38
LDL-C (mg/dL)	126±31	115±29	126±36	125±35	130±33	128±30	116±31
HDL-C (mg/dL)	44±12	41±12	60±16	54±15	32±6	31±6	46±12
TG (mg/dL)	140±88	152±98	128±71	130±80	167±120	165±70	136±55
DM (%)	8	11	5	7	24	24	NA

Numbers are average ± SD. CRP* was calculated as median. NA means no data was available.

**Table 2:** Percent distribution (%) of the four major CRP-molecular forms in the nine studied groups.

Group	CRPmf-1 (%)	CRPmf-2 (%)	CRPmf-3 (%)	CRPmf-4 (%)
FOS male control	26.5	32.4	25.8	8.7 [46]
FOS male CHD	22.7	33.2	29.1	9.4 [89]
FOS female control	22.4	32.9	27.9	8.1 [72]
FOS female CHD	25.9	37.4	23.3	8.8 [83]
VA-HIT no DM no Rx	17.5	31.6	25.2	22.1 [88]
VA-HIT no DM Rx	20.0	37.8	22.4	17.7 [90]
VA-HIT DM no Rx	19.5	31.4	24.3	17.2 [89]
VA-HIT DM Rx	17.1	38.4	23.9	14.3 [92]
Obese group (BMI> 60)	17.1	29.8	25.1	19.1 [100]

The numbers in brackets [] in the last column indicate the proportion of subjects with>5% of CRP-mf-4.

VA-HIT subjects, treated with either placebo or gemfibrozil, had a significantly higher proportion of CRP-mf-4 than FOS male controls (22.1% and 17.7% vs. 8.7%, respectively). In many samples, CRP-mf-4 was only a shoulder of CRP-mf-3. Therefore, we declared CRP-mf-4 as an independent particle only when the separation was clear and the signal was≥5% of total. Comparing male participants in the 2 groups we have seen significantly more subjects with≥ 5% of CRP-mf-4 in the VA-HIT (89%) than in the FOS controls (46%).

To investigate whether CVD significantly influenced CRP-mf distribution, we have compared CRP-mfs in FOS cases and controls, but have not seen significant differences in the distribution of the CRP-mfs between cases and controls. As there was significantly more type-2 diabetes (DM) in the VA-HIT than in the FOS cohort we also have tested whether DM was a confounder in the distribution of CRP-mfs using subjects with and without diabetes from the VA-HIT cohort. The analyses showed no significant association between DM and CRP-mfs.

Substantially more subjects with BMI≥30 than BMI ≤25 had CRP-mf-4 (88% vs. 38%, respectively). To verify this result we have done a series of comparisons. First, we compared CRP-mfs between non-obese (BMI ≤25) subjects with and without CVD in the FOS. In both groups, about the same percentile of subjects (38% vs. 42%) had CRP-mf-4 in their plasma. Next, we compared obese subjects (BMI≥30) with and without CVD with similar results to non-obese subjects (83% vs. 87%, respectively). It became obvious that significantly more obese than non-obese subjects had CRP-mf-4 (85% vs. 40%, respectively). Based on that result, we included 60 morbidly obese (BMI≥40) otherwise healthy subjects into the analyses. While about 40% of subjects with BMI ≤25 had CRP-mf-4, 100% of the severely obese subjects had CRP-mf-4 present in their plasma (**Table 2**).

Plasma CRP level and distribution of CRP-mfs had no significant association. Moreover, we have investigated subjects who had significant changes (10-50-fold) in CRP levels, probably due to an acute infection at one sampling-time point, but their CRP-mf distribution did not change with their CRP levels. We have tested whether any of the lipoprotein-associated parameters (TC, LDL-C, HDL-C, TG, apoA-I, apoB, Lp(a), and HDL and LDL subclasses) were associated with CRP-mfs, but only BMI and waist circumference showed significant associations. However, the association between obesity measurements and CRP-mf-4 was not continuous. Independent of actual BMI, obese subjects had higher levels of CRP-mf-4 than non-obese subjects. Neither CRP concentration nor the distribution of CRP-mfs and the presence of CRP-mf-4 changed significantly in a group of severely obese subjects (n = 20) after gastric bypass surgery, which resulted in a loss of 70 pounds of body weight, but still left subjects in the obese range (BMI>30) [13]. We have also compared CRP-mfs in 300 control female and 500 control male subjects from the FOS and found no gender difference in the CRP-mf distribution after adjusting data for BMI.

## Discussion

It is documented that native CRP (nCRP), a pentameric form, and modified CRP (mCRP), a monomeric form, are different in physiologic functions [14], [15]. To our knowledge, we are the first to report CRP-mfs in human plasma, which are different from the previously reported nCRP and mCRP. We have used non-denaturing gradient PAGE for separating CRP-mfs from human plasma. Since non-denaturing gradient PAGE separates particles by mass over particle-charge ratio rather than purely by size, we cannot ascertain the true molecular weight of these CRP molecular forms. Yet it is highly probable that the faster a CRP particle runs in a 3-16% gradient gel the smaller its size is. Actually the monomer CRP standard (25 KD) had the highest electrophoretic mobility and migrated ahead of albumin very close to the free-dye front. CRP-mf-5, the fastest CRP-mf, migrated behind albumin indicating that its molecular weight is> 70 KD and probably is a pentameric CRP. None of the three different monoclonal antibodies against mCRP reacted with any of the described CRP-mfs supporting our finding on the gel that none of the CRP-mf is a monomer.

*In vitro* and *in vivo* studies using purified CRP indicated several interactions of CRP with a variety of proteins participating in innate immunity [16]-[18]; however, we have not found IgG, IgM, C3, C4a, C4b and clusterin signals overlapping the CRP bands separated from whole plasma indicating that CRP-mfs are not part of an activated complement system. We have not seen significant associations between CRP levels and CRP-mfs demonstrating that the inflammatory response probably plays no significant role in the formation of these CRP-mfs.

We hypothesize that the observed CRP-mfs are assembled in the circulation by native CRP pentamers which stack similarly to serum amyloid protein (SAP) lattices in the aqueous phase. However, in contrast to SAP, CRP assembly into various molecular forms is not calcium-dependent. Alternatively, CRP might be bound to other proteins to form the various size CRP-mfs, but we have not been able to identify any such proteins despite immunoblotting with antibodies against apolipoproteins, immunoglobulins and complement factors.

It should be noted that in the absence of calcium and small molecule ligand(s), even at physiologic pH and ionic strength, CRP tends to aggregate. Although we separated CRP-mfs from human plasma, we do not know how Ca^2+^ concentration changes locally in the gel during electrophoresis. In spite of this uncertainty, we believe that our findings are physiologic and are supported by the consistency of finding these molecular forms in a large number of human plasma samples (1800 plasma samples separated under identical conditions).

The significant association between CRP-mf-4 and obesity suggests that fat cells release some factor(s), which controls the formation of CRP-mfs. We have not seen any difference in the distribution of CRP-mfs in obese subjects having primarily abdominal or subcutaneous fat. Although the liver has been assumed to be the lone source of CRP synthesis, some researchers believe that CRP is synthesized in adipose tissue as well [19]. Moreover, roughly 30% of circulating IL-6, the major regulator of CRP synthesis, is produced by adipose tissue. We have also examined a group of obese subjects with non-alcoholic fatty liver disease (NAFLD) whose liver-fat was measured (data not shown). Subjects with NAFLD usually have increased macrophage infiltration into liver tissue and increased inflammatory response as measured by increased IL-6 level. We have not seen any correlation between liver-fat and CRP-mf-4 after adjusting data for BMI. It should be noted that all subjects with NAFLD were obese; therefore the correlation between fatty liver and CRP-mf-4 might have been masked.

At neutral pH, 2 mM Ca^2+^, and 140 mM NaCl, both SAP and CRP exist in a pentamer-decamer equilibrium [20]. In the presence of calcium and in the absence of a ligand, long stacks of SAP pentamers have been shown to occur by electron microscopy [21].

Interestingly, we have not seen multiple-size SAP in human plasma separated by non-denaturing PAGE. SAP displayed only one band, which ran slightly above CRP-mf-5. We hypothesize that similar to SAP, CRP can form stacks of pentamers. This is supported by our observations on CRP-mf-1, which migrated in the VLDL and LDL size range (up to 1 million KD) and CRP-mf-2, which migrated in the large-HDL size range (about 300-500 KD). It is worth noting that CRP binding to LDL and HDL has been discussed for more than 20 years [22]-[26]. In contrast to others' reports [25], our own immunoblotting experiments using specific antibodies for apoB, apoA-I, and CRP indicated no CRP binding to LDL or HDL under physiologic conditions in human plasma. In our view, it is only purified and immobilized CRP that binds to LDL. Nunomura reported that immobilized CRP binds to LDL by attaching to apoB at the PC-binding site of CRP molecule [25]. Taskinen reported that purified CRP can bind to cholesterol via the 3-beta-hydroxyl group [26]. In contrast to these investigators, we have separated CRP-mfs from whole plasma in one step on native polyacrylamide gel, and we have not detected any significant binding of CRP to LDL or HDL under non-denaturating physiologic conditions.

We therefore assume that in plasma under physiologic ion concentrations and in the absence of a ligand, CRP forms a lattice-like structure similar to SAP. We also hypothesize that fat cells produce some factors that directly or indirectly influence CRP oligomerization.

## Conclusion

We have documented the consistent presence of 3 CRP molecular-forms (CRP mf-1, CRP mf-2, and CRP mf-3) in all 1800 human plasma samples that we have examined. We have also indentified one molecular form (CRP mf-4) which is strongly linked to human obesity. We have also shown that oligomerization of CRP is not calcium dependent and can be influenced by a yet unknown factor that is probably released from fat cells. Future research is required to further characterize these CRP molecular forms and to precisely ascertain their function and pathophysiologic significance. We hypothesize that adipose tissue produces a factor which influences the formation of CRP mf-4. Therefore, CRP-mfs might be used as an obesity-associated inflammatory marker.
